# Imprinting in plants as a mechanism to generate seed phenotypic diversity

**DOI:** 10.3389/fpls.2014.00780

**Published:** 2015-01-27

**Authors:** Fang Bai, A. M. Settles

**Affiliations:** Horticultural Sciences Department and Plant Molecular and Cellular Biology Program, University of FloridaGainesville, FL, USA

**Keywords:** epigenetics, DNA methylation, histone modification, imprinting, genomics, seed development, maize endosperm, *Arabidopsis* endosperm

## Abstract

Normal plant development requires epigenetic regulation to enforce changes in developmental fate. Genomic imprinting is a type of epigenetic regulation in which identical alleles of genes are expressed in a parent-of-origin dependent manner. Deep sequencing of transcriptomes has identified hundreds of imprinted genes with scarce evidence for the developmental importance of individual imprinted loci. Imprinting is regulated through global DNA demethylation in the central cell prior to fertilization and directed repression of individual loci with the Polycomb Repressive Complex 2 (PRC2). There is significant evidence for transposable elements and repeat sequences near genes acting as cis-elements to determine imprinting status of a gene, implying that imprinted gene expression patterns may evolve randomly and at high frequency. Detailed genetic analysis of a few imprinted loci suggests an imprinted pattern of gene expression is often dispensable for seed development. Few genes show conserved imprinted expression within or between plant species. These data are not fully explained by current models for the evolution of imprinting in plant seeds. We suggest that imprinting may have evolved to provide a mechanism for rapid neofunctionalization of genes during seed development to increase phenotypic diversity of seeds.

## OVERVIEW OF ANGIOSPERM SEED DEVELOPMENT

**FIGURE 1:**
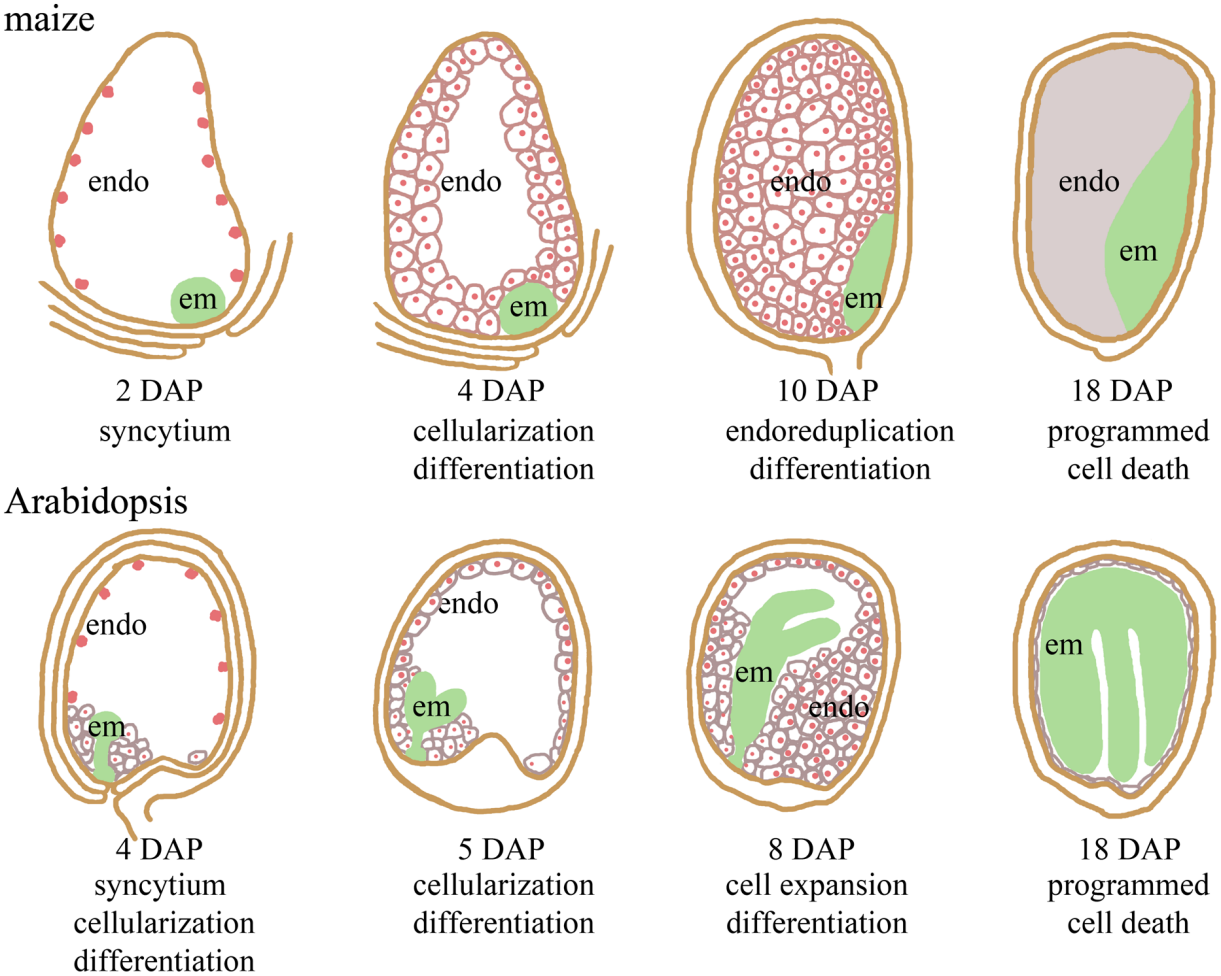
**Comparison of seed development in maize and *Arabidopsis thaliana*.** The endosperm proliferates initially as a multi-nucleate syncytium, while the globular embryo develops. Endosperm cellularization precedes embryo transition. Endosperm cells expand and accumulate storage molecules once cellularization is complete. In maize, the endosperm is persistent and undergoes programmed cell death starting around 18 days after pollination (DAP). The *Arabidopsis* embryo consumes most of the endosperm prior to seed maturation. Endosperm (endo) nuclei are indicated in red. The embryo (em) is in green.

In this review, we focus on the developmental role of epigenetic regulation, specifically genomic imprinting, in maize and *Arabidopsis* seeds. Imprinting, or parent-of-origin specific gene expression, has evolved convergently in mammals and angiosperms (Pires and Grossniklaus, 2014). Imprinted gene expression in angiosperms is found in developing seeds. Angiosperm seeds initiate with double fertilization of the megagametophyte (Peris et al., 2010). The pollen tube delivers two haploid sperm cells to the embryo sac. One sperm cell fuses with the haploid egg to generate a diploid embryo, and the other sperm cell fuses with the diploid central cell to form the triploid endosperm. The resulting embryo and endosperm are genetically identical except for their ploidy level with the endosperm having two maternal doses of the genome and one paternal dose. Although the endosperm and embryo have essentially the same genotype, they have markedly different developmental programs (**Figure 1**; Kiesselbach, 1949; Brown et al., 1999; Chandler et al., 2008; Peris et al., 2010).

The endosperm starts development by dividing nuclei without completing cytokinesis (reviewed in Olsen, 2004; Sabelli and Larkins, 2009b). This syncytial development transitions to cellularization in which the nuclei become enclosed in cell walls (**Figure 1**). As the endosperm cellularizes, the cells begin to take on differentiated fates with internal endosperm cells accumulating nutrient storage reserves (Kiesselbach, 1949; Brown et al., 1994, 1996, 1999; Stangeland et al., 2003). In many eudicots, like *Arabidopsis*, the embryo consumes the endosperm reserves as it develops resulting in most of the endosperm degenerating by seed maturity. By contrast, the internal storage cells in the maize endosperm persist through seed development and the storage reserves are used during seedling growth (Kiesselbach, 1949). Epidermal endosperm cells take on different fates from the internal storage cell types. In *Arabidopsis*, there are distinct endosperm cell morphologies at the micropyllar and chalazal ends of the embryo sac (Brown et al., 1999). In maize, epidermal endosperm cells differentiate into basal transfer cells, embryo surrounding region, and aleurone (Kiesselbach, 1949). All maize endosperm cells, except the aleurone, undergo programmed cell death prior to seed maturation (**Figure 1**; Young et al., 1997; Young and Gallie, 2000).

Embryo development starts with asymmetric cell division of the zygote to form an apical-basal axis (Chandler et al., 2008; Peris et al., 2010). Basal cells divide to develop the suspensor and contribute to the root meristem. Apical cells initially develop a globular embryo, which transitions to form the shoot and root apical meristems along with cotyledons in *Arabidopsis* or a scutellum, coleoptile, and embryonic leaves in maize (i.e., transition stage). The genetic programs controlling meristem specification and lateral organ initiation have been extensively reviewed (Chandler et al., 2008; De Smet et al., 2010; Wendrich and Weijers, 2013).

Imprinted genes primarily show parent-of-origin expression patterns in the endosperm although there are imprinted genes also in the developing embryo (Jahnke and Scholten, 2009; Raissig et al., 2013). Endosperm growth has a significant impact on final seed size, and imprinting has been hypothesized to regulate seed size (Arnaud and Feil, 2006; Xiao et al., 2006; Li and Berger, 2012; Fatihi et al., 2013). However, there is significant data arguing that the endosperm has developmental functions beyond providing nutrition for the developing embryo. Embryo transition occurs soon after endosperm cell differentiation, and recent evidence indicates differentiated endosperm is important for embryo developmental programs. For example, the embryo surrounding endosperm in *Arabidopsis* secretes the ESF1 signaling peptide to promote normal basal embryo development (Costa et al., 2014). Failure to differentiate the embryo surrounding endosperm in maize causes an embryo developmental block at the transition stage suggesting a similar function for this cell type in maize (Fouquet et al., 2011). Later in *Arabidopsis* seed development, the ZHOUPI basic-helix-loop-helix transcription factor is expressed exclusively in the embryo surrounding region and activates a signaling pathway required for normal epidermal differentiation in the embryo (Yang et al., 2008; Xing et al., 2013). These data show that the endosperm plays an active role in promoting embryo development and argue that epigenetic regulation of endosperm gene expression could have consequences for seed size as well as embryo developmental programs.

## WHAT IS IMPRINTING?

Genomic imprinting in plants is an epigenetic phenomenon by which genetically identical alleles are differentially expressed in a parent-of-origin dependent manner. Imprinted gene expression primarily occurs in the endosperm and there is strong data for imprinted genes controlling early endosperm cell divisions as well as regulating the transfer of nutrients to the seed (Gutierrez-Marcos et al., 2004; Day et al., 2008; Sabelli and Larkins, 2009a; Tiwari et al., 2010; Shirzadi et al., 2011; Costa et al., 2012). Imprinting is an exception from Mendel’s Laws on the expression and inheritance of the two parental alleles in which dominant alleles express phenotypes over recessive alleles irrespective of the parental source of the allele. Instead, imprinted genes will express either the maternal or paternal allele even though the primary sequences of these alleles may be identical.

**FIGURE 2:**
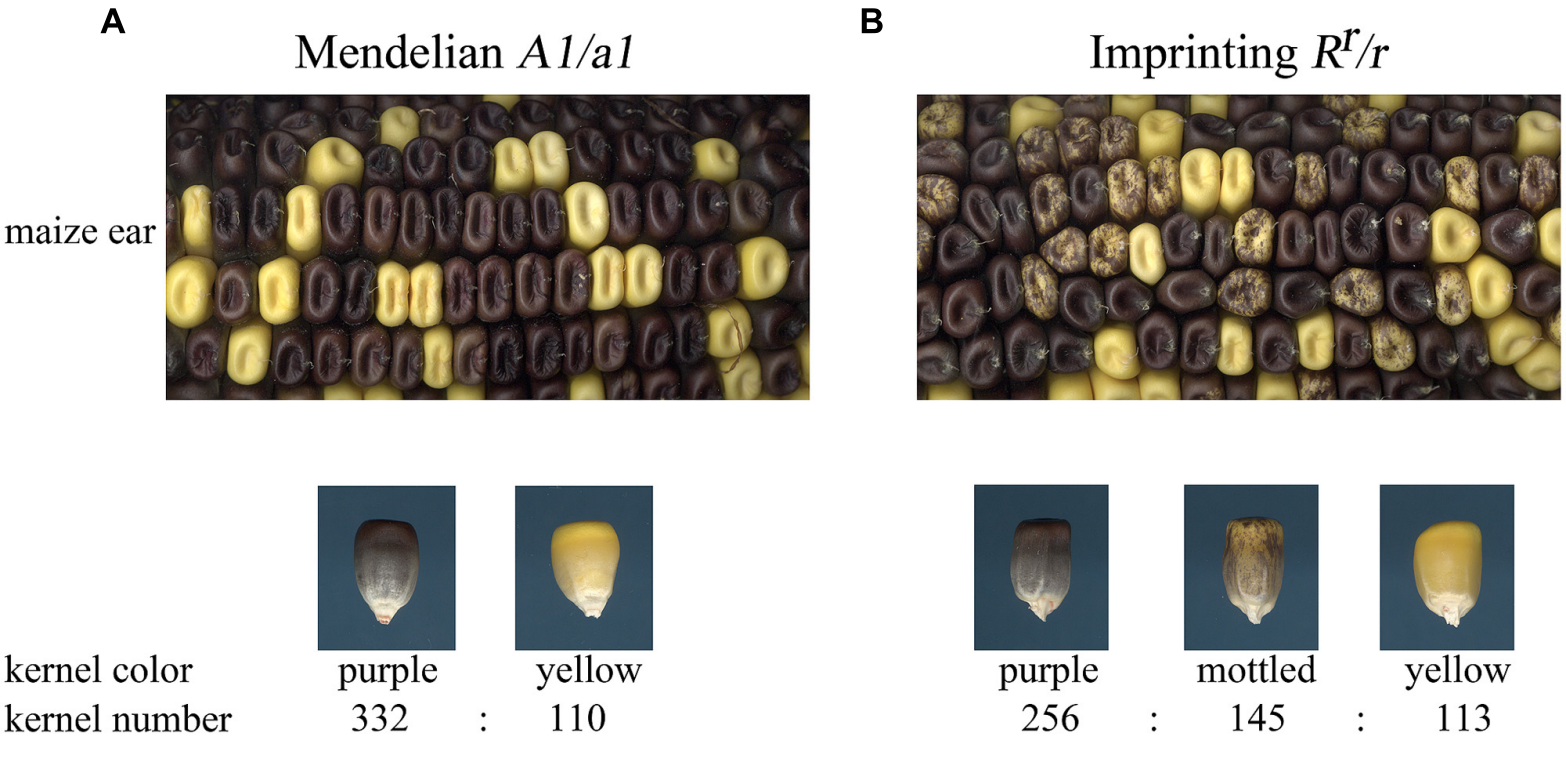
**Comparison of Mendelian genetic inheritance and imprinted inheritance. (A)** The *a1* locus shows Mendelian inheritance in self-pollinations of *A1/a1* individuals. Full purple color kernels are dominant over yellow kernels and the progeny segregate in a 3:1 ratio. **(B)** The *R^r^* allele shows imprinted inheritance. Self-pollination of *R^r^/r* yields three kernel color types in a 2:1:1 ratio of purple to mottled to yellow kernels. Purple kernels inherited the *R^r^* allele from the megagametophyte. Mottled kernels are heterozygous individuals that inherited the *R^r^* allele from the pollen. Kernel counts are given for the ears shown in the upper panels.

It is easiest to understand imprinted inheritance through an example. The *A1* locus of maize encodes a structural gene for anthocyanin biosynthesis (O’Reilly et al., 1985), while the *R* locus encodes a transcription factor that induces anthocyanin biosynthesis (Ludwig et al., 1989; Perrot and Cone, 1989). The *A1* locus shows Mendelian inheritance, while certain haplotypes of the *R* locus are imprinted (Kermicle, 1969). Indeed, *R* was the first imprinted locus described in plants. The *R^r^* allele shows altered expression when *R^r^* is inherited from pollen. The expression pattern of paternally inherited *R^r^* can be seen by contrasting self-pollinations of heterozygous individuals for *A1/a1* or *R^r^/r* (**Figure 2**). When a plant heterozygous for *A1/a1* is self-pollinated, the seeds segregate in a 3:1 ratio for full color to yellow kernels (**Figure 2**). Self-pollination of *R^r^/r* yields three kernel color types in a 2:1:1 ratio of purple to yellow to mottled purple kernels. These mottled purple kernels have an endosperm genotype of *r r/R^r^* where the dominant allele inherited from the male is repressed in a stochastic pattern in the seed. The same paternal *R^r^* allele is not affected in the embryo and plants from mottled kernels will yield full color kernels if crossed as female to an *r/r* plant and mottled kernels if crossed as a male to the *r/r* genotype.

One interpretation of the mottled *r r/R^r^* kernel phenotype is that it is due to an insufficient dosage as a consequence of the endosperm fusing a diploid maternal central cell with a haploid paternal sperm cell. However, introducing multiple copies of *R^r^* with translocation and trisomic stocks does not alter the anthocyanin phenotypes. When more than two doses are inherited maternally, the kernel is always full color, and when multiple *R^r^* alleles are inherited paternally, the mottled phenotype always results (Kermicle, 1970). These and more recent data indicate that imprinting is an independent phenomenon from dosage effects.

The *r* locus is an example of maternal bias of gene expression. Imprinted genes can show bias for either the maternal or paternal allele and consequently are classified into maternally expressed genes (MEGs) and paternally expressed genes (PEGs). MEGs and PEGs can be identified molecularly by examining allele-specific expression in reciprocal crosses. Single nucleotide polymorphisms and small insertion-deletions within transcripts from diverse parents are used to identify the expression level of both the maternal and paternal allele. By carrying out the same gene expression analysis on reciprocal crosses, it is possible to identify genes that express only the maternal or only the paternal allele irrespective of the polymorphisms found within the alleles of the genes. A variety of molecular strategies have been employed to identify individual imprinted genes such as *Maternally expressed gene1* (*Meg1*) and *Maternally expressed in embryo1* (*Mee1*) in maize (Gutierrez-Marcos et al., 2004; Jahnke and Scholten, 2009).

RNA-seq transcriptomics allows global analysis of imprinted gene expression at a much larger scale. Maize is particularly well suited for allele identification in RNA-seq experiments, because maize inbred lines show high levels of polymorphism allowing for a large number of genes to be assayed for imprinting in a single experiment (Chia et al., 2012; Jiao et al., 2012). Initial experiments in maize examined reciprocal crosses between the reference genome inbred line, B73, and Mo17 (Waters et al., 2011; Zhang et al., 2011). These studies identified hundreds of MEGs and PEGs with relatively little overlap between them. By examining additional time-points during seed development and additional inbred crosses more than 500 genes show statistically significant bias for gene expression based on the parent of origin (Waters et al., 2013; Zhang et al., 2014). Many imprinted genes only show parent-of-origin bias transiently. For example, *Meg1* is maternally expressed early during seed development and is biallelic, expressed from both maternal and paternal alleles, by mid-seed development (Gutierrez-Marcos et al., 2004). Attempts to apply transcriptomics to early stages of developing maize seeds did not effectively isolate endosperm or embryo tissue from maternal tissue, so most identified maize MEGs and PEGs show imprinted expression patterns after embryo transition (Xin et al., 2013). The overlap of imprinted genes between all data sets is low. For example, Waters et al. (2013) found that only 5–10% of the imprinted genes in a survey of four inbred lines showed imprinting in all genotype combinations. These results have raised questions about whether sequencing depth, statistical approaches, allele-specific effects, or environmental factors have significant impact on the genes detected as imprinted.

Similar transcriptomic approaches have been applied to identify imprinted genes in *Arabidopsis*. Endosperm and embryo transcripts from reciprocal crosses between *Col-0* and *Ler* accessions identified over 200 imprinted genes (Gehring et al., 2011; Hsieh et al., 2011). Expanding these studies to additional accessions found that, like in maize, only a small number of genes consistently show imprinting in all accessions (Wolff et al., 2011; Pignatta et al., 2014). For example, Pignatta et al. (2014) found about 10% of MEGs and 5% of PEGs are shared between the three accessions they surveyed. It seems surprising that both maize and *Arabidopsis* transcriptome surveys have found only a few conserved imprinted genes within each species. Considering both the maize and *Arabidopsis* observations, the data suggest that relatively few loci are stably selected for imprinted gene expression.

Transcriptomic studies have also identified allele-specific imprinting in both maize and *Arabidopsis* (Waters et al., 2013; Pignatta et al., 2014). Allele-specific imprinted genes have MEG or PEG expression for a single allele from a single accession or inbred line, much like the *R^r^* allele of maize. Using kernel phenotypes, allele-specific imprinting has been observed in other maize loci including the *dzr1^Mo17^* and *B-Boliva* alleles that control zein and anthocyanin accumulation, respectively (Chaudhuri and Messing, 1994; Selinger and Chandler, 2001). These older examples indicate that allele-specific imprinting can have significant effects on kernel phenotypes. However, phenotypes have not been associated with the more recently discovered allele-specific imprints.

With hundreds of imprinted genes identified, annotation of these genes suggests that imprinted loci do function in processes proximate to developmental programs. Imprinted genes include proteins involved in chromatin modification, transcription factors, hormone signaling, ubiquitin-targeted protein degradation, and RNA processing (Gehring et al., 2011; Hsieh et al., 2011; Wolff et al., 2011; Zhang et al., 2011; Xin et al., 2013; Pignatta et al., 2014). MEGs show some enrichment for transcription factors, such as MYB family genes (Hsieh et al., 2011; Pignatta et al., 2014), while PEGs show enrichment for chromatin and transcriptional modifiers (Waters et al., 2013; Pignatta et al., 2014). However, only two genes show conserved imprinted expression between *Arabidopsis*, rice, and maize (Waters et al., 2013). A loss of function allele of one of these PEGs, *ZmYuc1*, is tightly linked to the recessive *defective endosperm18* locus of maize, suggesting that residual maternal expression is sufficient to confer normal seed development (Bernardi et al., 2012). Both allele-specific imprinting and the low conservation of imprinted gene expression across angiosperms suggest that deeply conserved developmental circuits have not been selected for this type of epigenetic regulation in angiosperms. Based on these and additional arguments below, we suggest that imprinting is primarily a form of regulation to enable rapid diversifying selection of seed phenotypes.

## MOLECULAR MECHANISMS OF IMPRINTING

Altering the expression state of an allele depending upon the parent-of-origin requires epigenetic modification of the alleles inherited by the male and female gametes. The mechanisms by which MEGs and PEGs are identified and programmed have been extensively reviewed (Köhler et al., 2012; Gehring, 2013; Zhang et al., 2013). As a brief overview, both histone modification and DNA methylation have essential roles in setting imprinted patterns of gene expression. The *Arabidopsis* model for establishing contrasting epigenetic states in the male and female gametes starts with differential demethylation of the genome. The DNA glycosylase gene, *DEMETER (DME)*, is expressed in the central cell of the megagametophyte but not the sperm cells of the pollen (Choi et al., 2002; Schoft et al., 2011). *DME* activity removes 5-methylcytosine predominantly from transposable element and repeat sequences leading to most repetitive sequences having reduced methylation in the developing endosperm (Gehring et al., 2009; Hsieh et al., 2009). Surprisingly, maize does not show these global patterns of DNA hypomethylation in the endosperm (Zhang et al., 2011, 2014). Instead, allele-specific bisulfite sequencing of endosperm DNA revealed a pattern of DNA hypomethylation at maternal alleles with corresponding hypermethylation at paternal alleles for specific sites within the genome (Zhang et al., 2014). These maize results are consistent with DNA demethylation specifically occurring in the central cell.

The differential loss of DNA methylation sets-up contrasting chromatin marks in *Arabidopsis* repeat sequences near the paternal and maternal alleles. Methylation marks can then be interpreted by the genome with a variety of molecular mechanisms. For example, methylation of the paternal allele can lead to a transcriptionally silent state, while the demethylated maternal allele would become transcriptionally active (Kinoshita et al., 2004; Jullien et al., 2006a; Hermon et al., 2007; Tiwari et al., 2008). There are also a few examples where RNA-directed DNA methylation (RdDM) is critical in the male parent to ensure silencing of the paternal allele at MEG loci, suggesting that small RNAs can have a significant role in setting MEG expression patterns (Bratzel et al., 2012; Vu et al., 2013). Although these models can explain MEG patterns of expression, PEGs can also be hypermethylated at the paternal allele and hypomethylated at the maternal allele (Gehring et al., 2009; Hsieh et al., 2009; Zhang et al., 2014). This maternal hypomethylation is essential for silencing of the maternal allele for many PEGs (Hsieh et al., 2011; Wolff et al., 2011).

How can the same epigenetic mark of reduced DNA methylation in the maternal allele result in opposite MEG and PEG expression patterns? Trimethylation of lysine 27 on histone H3 (H3K27me3) is another chromatin mark that is required for imprinted gene expression (Schuettengruber and Cavalli, 2009; Köhler et al., 2012). H3K27me3 marks are catalyzed by the Polycomb Repressive Complex2 (PRC2). In the *Arabidopsis* endosperm, the PRC2 complex is referred to as the FERTILIZATION INDEPENDENT SEED (FIS) complex and is composed of four core subunits: the MEDEA (MEA) Enhancer of zeste homolog, the FERTILIZATION INDEPENDENT SEED2 (FIS2) Suppressor of zeste homolog, the FERTILIZATION INDEPENDENT ENDOSPERM (FIE) Extra sex combs homolog, and the MULTICOPY SUPPRESSOR OF IRA1 (MSI1), which is a WD-40 repeat protein that is homologus to Drosophila p55 (Grossniklaus et al., 1998; Kiyosue et al., 1999; Luo et al., 1999; Köhler et al., 2003). The H3K27me3 post-translational modification is a repressive chromatin mark, and FIS-PRC2 is known to be required to repress paternal alleles of MEGs as well as maternal alleles of PEGs (Köhler et al., 2003, 2005; Baroux et al., 2006; Makarevich et al., 2006; Fitz Gerald et al., 2009; Weinhofer et al., 2010).

It is not inherently obvious how PRC2 would differentially target hypo- or hypermethylated DNA. PRC2 is recruited to cis-elements at repressed loci. These PRC2 recruitment elements have been identified in multiple organisms and can include repeat sequences (Kinoshita et al., 2007; Makarevich et al., 2008), small segments of CG-rich sequence (Jermann et al., 2014), or transcription factor binding sites (Berger et al., 2011; Liu et al., 2011; Lodha et al., 2013). In addition, non-coding RNA has been shown to interact with PRC2 and target it to specific loci in plants (Heo and Sung, 2011). DNA methylation interferes with PRC2 function and prevents H3K27me3 modification (Weinhofer et al., 2010; Deleris et al., 2012; Jermann et al., 2014). Thus, hypermethylation of paternal alleles can interfere with PRC2 recruitment sites allowing expression of the paternal allele, while PRC2 activity at the maternal, hypomethylated allele would result in transcriptional silencing to give a PEG pattern of expression. Global analysis of H3K27me3 sites in maize supports this model (Makarevitch et al., 2013). Indeed, Zhang et al. (2014) found that PEGs showed enrichment for maternal H3K27me3 marks concomitant with hypomethylation at the maternal allele and hypermethylation at the paternal allele.

PRC2 is also required to repress the paternal allele of some MEGs (Baroux et al., 2006; Gehring et al., 2006; Jullien et al., 2006b). However, there is no molecular mechanism proposed for how PRC2 would preferentially target the hypermethylated, paternal allele at a MEG locus. Maternal specific expression is also observed for the *Arabidopsis ZIX* locus, but the MEG pattern of expression does not dependent upon DME or FIS-PRC2 (Ngo et al., 2012). Moreover, imprinted gene expression is documented within the embryo of both maize and *Arabidopsis* (Jahnke and Scholten, 2009; Raissig et al., 2013). The DME DNA glycosylase is not expressed significantly in the *Arabidopsis* egg cell (Choi et al., 2002), and there is no evidence for global DNA demethylation in the embryo (Hsieh et al., 2009). The FIS-PRC2 complex is required for some embryo imprinted gene expression suggesting that H3K27me3 does have a functional role in setting-up MEG and PEG expression in the embryo (Raissig et al., 2013). These observations indicate that we are far from completely understanding the molecular mechanisms guiding imprinted expression patterns during seed development.

## IS IMPRINTING NECESSARY FOR SEED DEVELOPMENT?

Genetic analysis of imprinted genes suggests a similar spectrum of developmental functions as for biallelic-expressed genes. As mentioned earlier, imprinting in maize can affect non-essential genes regulating anthocyanin biosynthesis or storage protein accumulation. There are also numerous imprinted genes that have been shown to have critical roles in seed development. Superficially, it is simple to conclude that imprinted expression patterns are therefore critical to seed development. However, we argue that most of these examples fail to provide conclusive evidence that the imprinted pattern is indispensable as opposed to a minimum expression level of the gene being critical.

The* MEA, FIE,* and *FIS2* genes encode subunits of PRC2 and are MEGs in *Arabidopsis* (Luo et al., 2000). Loss-of-function mutations in these genes have profound effects on seed development with 50% seed abortion, delays in endosperm and embryo development, and increased cell proliferation in the developmentally delayed endosperm and embryo (Ohad et al., 1996; Chaudhury et al., 1997; Grossniklaus et al., 1998; Kiyosue et al., 1999). Moreover, these mutants can begin central cell divisions even when the ovule is not fertilized. The FIS-PRC2 phenotypes have been interpreted as imprinted expression of PRC2 being a key repressor of seed growth. However, very similar phenotypes are observed in mutants of the non-imprinted subunit of the FIS-PRC2 complex, *MSI1*, suggesting that imprinted gene expression is not directly responsible for the seed phenotypes (Köhler et al., 2003; Guitton and Berger, 2005; Leroy et al., 2007). This conclusion is further supported by the maize *MEA/Enhancer of zeste* (*Mez1*) gene, which is an endosperm MEG (Haun et al., 2007). A transposon insertion in the promoter region of the locus causes biallelic expression of *Mez1* but no change in seed phenotype (Haun et al., 2009). These data show that FIS-PRC2 function needs to be expressed at sufficient levels in both the female gametophyte and developing seed. However, direct evidence is lacking to support the hypothesis that imprinted expression of the PRC2 subunits is required.

The* PHERES1* (*PHE1*) gene is consistently up-regulated in *Arabidopsis mea, fie*, and *fis2* mutants (Köhler et al., 2003). The *PHE1* locus was the first PEG to be identified and encodes the AGAMOUS-LIKE37 (AGL37) MADS-domain protein that is a predicted transcription factor (Köhler et al., 2003, 2005). Knocking-down expression of *PHE1* in a *mea* mutant can partially rescue *mea* defective seed phenotypes suggesting that part of the FIS-PRC2 mutant phenotypes are due to increased *PHE1* expression (Köhler et al., 2003). However, insertion mutants in the 3′ regulatory region of *PHE1* can cause a loss of imprinting, switching to a biallelic pattern, with no effect on seed phenotype reported (Makarevich et al., 2008). *PHE1* is one of several *AGL* genes, including *AGL28*, *AGL36*, *AGL40*, *AGL62*, and *AGL90*, which are up-regulated when endosperm cellularization is delayed either by PRC2 mutants or genome dosage imbalances (Kradolfer et al., 2013a). Although *AGL28* and *AGL36* are MEGs (Shirzadi et al., 2011; Wolff et al., 2011), the other *AGL* genes are expressed from both parental alleles. Mutations in *AGL62* cause recessive seed defects illustrating that FIS-PRC2 complex influences biallelic genes as well as imprinted genes (Kang et al., 2008). Total expression levels of the *AGL* co-expression network correlates well with the timing of endosperm cellularization and embryo development (Walia et al., 2009; Tiwari et al., 2010; Kradolfer et al., 2013a). The divergent mechanisms of epigenetic control for these *AGL* genes and the lack of a requirement for paternal expression of *PHE1* suggest that imprinting *per se* is not likely the primary regulator of this developmental node.

An additional *Arabidopsis* PEG, *ADMETOS* (*ADM*), has been implicated in regulating the *AGL* gene node (Kradolfer et al., 2013b). *ADM* encodes a recently evolved J-domain protein that is only found in a few genera of the *Brassicaceae*. Consistent with its PEG expression, the *adm* locus was identified as a paternal-specific suppressor of seed abortion due to paternal genome excess (Kradolfer et al., 2013b). When mutated, *adm* reduces the overexpression of *PHE1* and other *AGL* genes toward normal both in interploidy crosses and in *mea* mutants. Although *ADM* is a PEG in multiple *Arabidopsis* accessions (Hsieh et al., 2011; Wolff et al., 2011), natural variation reducing *ADM* expression level in the L*er* accession is correlated with improved seed development and viability in paternal genome excess crosses (Kradolfer et al., 2013b). When *adm* is mutant in both maternal and paternal gametes, *adm* more effectively reduces *AGL* expression as well as more effectively suppresses seed abortion due to either paternal genome excess or *mea*, suggesting the maternal allele expresses at a developmentally significant level (Kradolfer et al., 2013b). Interestingly, homozygous *adm/adm* plants, overexpression of *ADM*, and biallelic expression of *ADM* have no seed phenotype in diploid crosses, suggesting that imprinting of this gene is not necessary for normal diploid seed development. The wild-type function of *ADM* is primarily to block interploidy and interspecific hybridizations.

The *Arabidopsis FORMIN HOMOLOGUE5* (*AtFH5*) gene is a MEG in which the paternal allele is repressed by PRC2 (Fitz Gerald et al., 2009). ATFH5 is an actin nucleator and is critical for cell plate formation and endosperm cellularization (Ingouff et al., 2005). Ectopic expression of the paternal allele of *AtFH5* does not impact *mea* mutant phenotypes (Fitz Gerald et al., 2009), suggesting paternal silencing of *AtFH5* may not be required for normal endosperm development. Moreover, double mutants of *mea* and *atfh5* show additive endosperm cellularization and morphogenic defects (Fitz Gerald et al., 2009). These genetic results typically would be interpreted as indicating *mea* and *atfh5* act in different genetic pathways. Although *AtFH5* is clearly an imprinted gene with a critical endosperm development function, it is unclear whether the imprinted gene expression pattern has a significant role in endosperm development.

The role of imprinting for the *Arabidopsis MATERNALLY EXPRESSED PAB C-TERMINAL (MPC)* gene is even less clear than for *AtFH5*. *MPC* encodes the C-terminal domain of poly(A) binding proteins (PABP) and is hypothesized to have a role in regulating translation of mRNA (Tiwari et al., 2008). The *MPC* gene is a MEG, and homozygous* mpc* RNAi lines show abnormal embryo and endosperm development. However, the role of imprinted gene expression for *MPC* function is difficult to address, since the gene body sequence is necessary to confer maternal specific expression (Tiwari et al., 2008).

In maize, only one imprinted gene has been functionally characterized in seed development. The *meg1* locus encodes a small, secreted peptide that is expressed specifically in the basal endosperm transfer cell layer of the developing endosperm (Gutierrez-Marcos et al., 2004). *Meg1* is initially expressed from the maternal allele and becomes biallelic around 12 days after pollination. RNAi of *meg1* results in reduced transfer cell differentiation and smaller seeds than non-transgenic controls (Costa et al., 2012). Ectopic expression of *Meg1* results in patchy, ectopic transfer cell differentiation throughout the epidermal endosperm, indicating that MEG1 protein is a positive regulator of transfer cell fate.

*Meg1* is part of a gene family with six members expressing at significant levels in transfer cells during seed development (Xiong et al., 2014). Only *Meg1* shows imprinted expression with all other *Meg* family members expressing similarly when inherited through either parent. The developmental function of these other *Meg* family members has not yet been experimentally tested. However, the expression patterns of these genes are very similar to *Meg1* in both developmental timing and location, suggesting that these genes are also likely to be regulators of transfer cell differentiation (Xiong et al., 2014). To directly test the role of imprinting on *Meg1* function, Costa et al. (2012) developed a non-imprinted, synthetic *Meg1* gene with two different transfer cell specific promoters. These transgenics show a dosage-sensitive increase both in the number of transfer cells and in seed size suggesting that imprinting of *Meg1* serves to limit nutrient uptake and seed size. Thus, among more than a dozen seed developmental genes studied in detail, only *Meg1* has strong evidence indicating that imprinted gene expression has a significant impact on development and growth of the seed.

## WHY DOES IMPRINTING EXIST IN ANGIOSPERMS?

Parent-of-origin specific gene expression is a fascinating pattern of molecular regulation of the genome, and its evolution has been the subject of extensive theoretical debate (Patten et al., 2014). The most accredited explanation for imprinting in plants is provided by the parental-conflict hypothesis, also known as the kinship theory of selection (Haig and Westoby, 1989; Haig and Westoby, 1991; Haig, 2013). This hypothesis argues that imprinting evolves when the maternal parent provides resources during offspring development. In angiosperms, seeds require nutrition from the maternal parent from fertilization until seed maturation. The parental-conflict hypothesis states that the paternal genome expression is selected to increase support for individual progeny, while the maternal genome expression is selected to limit resources to maximize seed set.

The parental-conflict hypothesis predicts that MEGs should reduce seed size and potentially reduce seed set in unfavorable conditions. Conversely, PEGs would increase seed size and promote seed set. Loss-of-function phenotypes of the FIS-PRC2 mutants *mea, fis2, fie*, and *msi1* have been interpreted to support parental-conflict theory, because these mutants extend cell proliferation in the endosperm and embryo at the cost of failing to complete development (Grossniklaus et al., 1998; Kiyosue et al., 1999; Guitton and Berger, 2005; Ingouff et al., 2005). However, parental-conflict predicts stable networks of imprinted genes with MEGs and PEGs balancing each other for normal seed development (Patten et al., 2014). As discussed above, most imprinted genes, except for *Meg1*, that have been studied in detail can lose imprinted gene expression without significant consequence to seed development, suggesting MEG or PEG expression does not undergo significant selection pressure. The case of the *Meg1* gene also argues against the parental-conflict hypothesis. *Meg1* is normally maternally expressed, and a non-imprinted *Meg1* transgene shows a positive dosage effect for increasing seed size (Costa et al., 2012). The parental-conflict hypothesis would predict that a maternally expressed peptide like MEG1 should be a repressor of transfer cell development or that *Meg1* should be a PEG.

An alternate hypothesis to explain imprinting is the maternal-offspring coadaptation model of gene expression, in which maternal alleles may be selected for imprinted expression to provide the greatest combined fitness for the mother and offspring (Wolf and Hager, 2006). This model is meant primarily to explain the larger number of MEGs over PEGs that have been identified in both mammals and angiosperms. For *Meg1*, the model correctly predicts maternal specific expression, but maternal-offspring coadaptation does not explain the relatively extensive number of PEGs or the apparent mutability of most angiosperm imprinted genes to switch between biallelic and imprinted states (Patten et al., 2014).

More in-depth evolutionary analysis of the identified imprinted genes in *Arabidopsis* suggests that imprinting correlates with rapid evolution of gene duplicates. More than two-thirds of *Arabidopsis* imprinted genes derive from recent gene duplication events (Qiu et al., 2014). *Arabidopsis* imprinted genes also show reduced domains of expression and increased evolutionary rates over non-imprinted paralogs. This analysis argues strongly that imprinted genes are undergoing neofunctionalization. Neither the parental-conflict nor the coadaptation models predict that recent gene duplication events would be favored for imprinted expression, although the bias toward gene duplicates does not specifically argue against these evolutionary models (Patten et al., 2014).

The current understanding of cis-elements targeting genes for imprinting further suggests that imprinting is primarily a rapid form of evolution. Transposons and short repeats appear to be the targets of differential demethylation in the central cell versus sperm cells (Gehring et al., 2009; Hsieh et al., 2009). Transposon movement allows random conversion of genes to an imprinted pattern of expression. For example, differences in transposon insertions near genes are associated with allele-specific imprinting in *Arabidopsis* (Pignatta et al., 2014). Transposon and other insertions can also convert imprinted genes to biallelic expression patterns, providing a fast mechanism to revert alleles into Mendelian, diploid expression (Haun et al., 2009). Importantly, transposition is known to increase in plants exposed to abiotic and biotic stress, suggesting imprinted gene expression is expected to change more rapidly when plants are poorly adapted to an environment (reviewed in Chénais et al., 2012). Although transposon insertions are generally thought to reduce gene expression, genome-wide analysis of gene expression and DNA methylation of 140 *Arabidopsis* accessions suggests transposon insertion within genes is associated with increased expression levels specifically during seed and pollen development (Schmitz et al., 2013). This more permissive epigenetic state allows genes silenced in other tissues to be expressed during seed development.

Based on the recent genome-wide analyses of imprinting and epigenetic regulation, we suggest that imprinting is a form of epigenetic regulation that allows more rapid selection on recent gene duplicates. Imprinting uncovers individual alleles by converting genes into a pseudohaploid mode of expression during seed development. There is no evidence for prolonged, imprinted expression of genes after germination, and many imprinted genes are expressed later in plant development (Pignatta et al., 2014). Thus, imprinting of one copy of a gene duplicate enables the imprinted gene to accumulate mutations without compromising whole plant fitness. Monoallelic expression in the seed exposes an imprinted allele to more rapid selection acting primarily upon the seed phenotype. Imprinting of recessive, advantageous alleles can confer greater fitness if only expressed from one parent. By contrast, selection against deleterious imprinted alleles is not as strong as in true haploid inheritance. Deleterious imprinted alleles would only be selected against when inherited from the parent conferring expression. For example, a deleterious PEG would be neutral when inherited from the mother. If a deleterious PEG is linked to an advantageous MEG allele, it could be maintained in a population for a significant period, potentially allowing time for additional compensatory mutations. Thus, imprinted expression may allow plant genomes to explore a larger space of allelic and phenotypic variation in the seed while avoiding deleterious plant phenotypes. The mature seed phenotype is expected to be a major driver of species fitness, and we suggest imprinting is a form of gene expression that allows for more efficient diversifying selection on the seed phenotype.

An important consequence of hypothesizing imprinting as a form of diversifying selection is that most imprinted expression patterns would be expected to have neutral effects on the fitness of the seed. Gene networks that appear to fit parental-conflict or coadaptation models are expected to evolve under diversifying selection. However, the prediction is that the bulk of imprinted expression patterns could revert to biallelic expression with no consequence on seed phenotype. Similarly, allele specific imprinting and novel imprinted loci would be expected to evolve at high frequency. Additional functional data of imprinted genes in outcrossing species such as maize, would help resolve whether parental-conflict or other types of selection is the primary driving force for the evolution of imprinted genes.

## Conflict of Interest Statement

The authors declare that the research was conducted in the absence of any commercial or financial relationships that could be construed as a potential conflict of interest.
